# 2132. Surveillance of Eravacycline Against Enterobacterales and Non-Fermenter Clinical Isolates, Including Resistant Isolates, Collected Worldwide from Multiple Infection Sites During 2021

**DOI:** 10.1093/ofid/ofad500.1755

**Published:** 2023-11-27

**Authors:** Stephen Hawser, Nimmi Kothari, Federica Monti, Tony Hodges, Kristie Zappas

**Affiliations:** IHMA Europe, Monthey, Valais, Switzerland; IHMA, Monthey, Valais, Switzerland; IHMA, Monthey, Valais, Switzerland; La Jolla Therapeutics, Waltham, Massachusetts; Innoviva Specialty Therapeutics, Inc., Waltham, Massachusetts

## Abstract

**Background:**

Eravacycline is a fully synthetic, fluorocycline approved for the treatment of complicated intra-abdominal infections (cIAI) in patients ≥18 years of age in Europe, Singapore, Hong Kong, mainland China, the US, and the UK. The purpose of this study was to monitor the *in vitro* activity of eravacycline against 2,942 Gram-negative clinical isolates, including multidrug-resistant (MDR) isolates, collected worldwide in 2021.

**Methods:**

As part of a worldwide surveillance study, a total of 2,942 Gram-negative isolates were collected during 2021 from various infections including IAI, body fluids, respiratory and other sources. Of the 2,942 isolates, 654, 1263, and 1025 were from Asia/Pacific, Europe, and North America, respectively. Minimum inhibitory concentrations (MICs) were determined by CLSI broth microdilution. Antibiotic susceptibility was determined with FDA or CLSI breakpoints.

**Results:**

Summary MIC data for tested antimicrobials are shown in the Table. Susceptibility to eravacycline for Enterobacterales was 93.9%, 85.7%, 90.0%, and 96.8% for all isolates, MDR Enterobacterales, ESBL-positive, and ESBL-negative isolates, respectively. Although there are no clinical breakpoints for *Acinetobacter baumannii* or *Stenotrophomonas maltophilia*, eravacycline exhibited promising activity against these species with MIC_50_ / MIC_90_ of 0.5 & 1 and 1 & 2 µg/ml, respectively.

**Figure:**
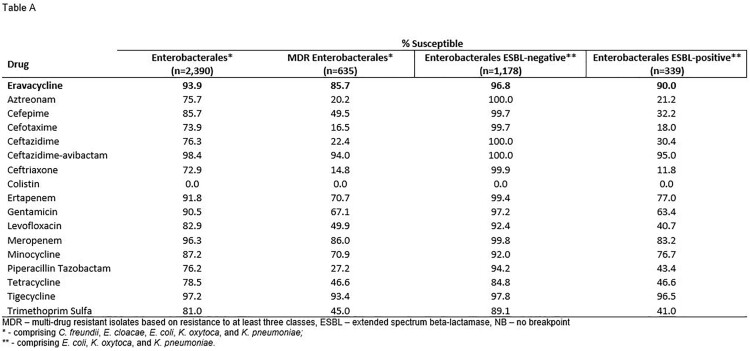

**Figure:**
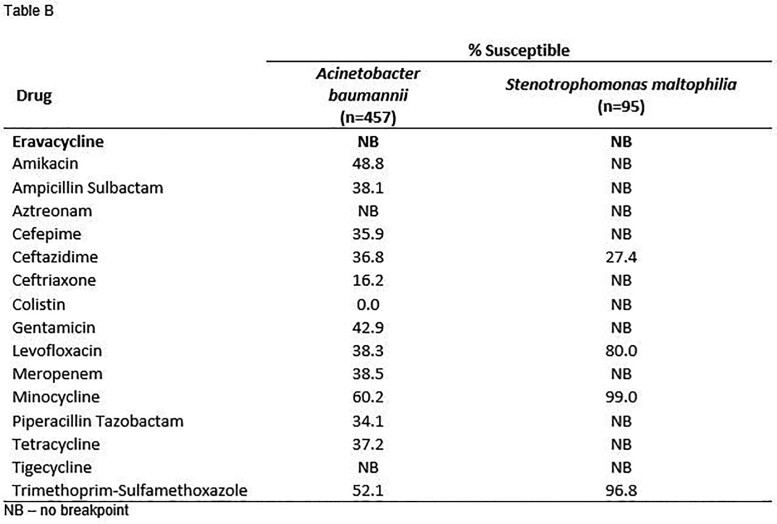

**Conclusion:**

Eravacycline exhibited sustained high susceptibility against multiple Enterobacterales species, including MDR and ESBL-positive isolates. While breakpoints are not defined for *A. baumannii* or *S. maltophilia*, eravacycline demonstrated promising activity against these species. These results suggest that, following several years of clinical use, susceptibility to eravacycline remains high.

**Disclosures:**

**Stephen Hawser, PhD**, Allecra: study funding|Innoviva Specialty Therapeutics, Inc.: Honoraria|Roche: Honoraria|Roche: This project has been funded by BARDA (HHSO100201600038C). **Nimmi Kothari, PhD**, Allecra: Allecra (study funding)|Innoviva Specialty Therapeutics, Inc.: Honoraria|Roche: Honoraria|Roche: This project has been funded by BARDA (HHSO100201600038C). **Federica Monti, PhD**, Allecra: Study funded|Innoviva Specialty Therapeutics, Inc.: Honoraria **Tony Hodges, MD**, La Jolla Pharmaceutical Company: Employee|La Jolla Pharmaceutical Company: Stocks/Bonds **Kristie Zappas, PhD**, La Jolla Pharmaceutical Company: employee|La Jolla Pharmaceutical Company: Stocks/Bonds

